# Correction to: A Comprehensive Study of Light Quality Acclimation in *Synechocystis* Sp. PCC 6803

**DOI:** 10.1093/pcp/pcaf049

**Published:** 2025-05-22

This is a correction to: Tomáš Zavřel, Anna Segečová, László Kovács, Martin Lukeš, Zoltán Novák, Anne-Christin Pohland, Milán Szabó, Boglárka Somogyi, Ondřej Prášil, Jan Červený, Gábor Bernát, A Comprehensive Study of Light Quality Acclimation in Synechocystis Sp. PCC 6803, *Plant and Cell Physiology*, Volume 65, Issue 8, August 2024, Pages 1285–1297, https://doi.org/10.1093/pcp/pcae062.

The authors noticed an error in the grant number listed in the `Funding' section of the published article, where it is mistakenly stated as:

The Ministry of Education, Youth and Sports of CR (LM2018123; CZ.02.1.01/0.0/0.0/16_026/0008413; LUAUS24131)

The correct information should, however, be:

The Ministry of Education, Youth and Sports of CR (LM2018123; CZ.02.1.01/0.0/0.0/16_026/0008413; LUAUS24149)

The error has now been corrected online in the published article.

